# Psychometric Properties of Pain Catastrophizing Scale in Patients with Carpometacarpal Osteoarthritis of Thumb—Item Response Theory Analysis

**DOI:** 10.3390/jcm15082835

**Published:** 2026-04-08

**Authors:** Sara Suomela, Mikhail Saltychev, Juhani Juhola, Hanna-Stiina Taskinen

**Affiliations:** 1 Department of Neurology, Turku University Hospital and University of Turku, 20521 Turku, Finland; 2 Department of Physical and Rehabilitation Medicine, Turku University Hospital and University of Turku, 20521 Turku, Finland; mikhail.saltychev@gmail.com (M.S.); juhani.juhola@varha.fi (J.J.); 3 Department of Orthopaedics and Traumatology, Turku University Hospital and University of Turku, 20521 Turku, Finland; hanna-stiina.taskinen@varha.fi

**Keywords:** catastrophizing, carpometacarpal joint, osteoarthritis, psychometrics, pain management

## Abstract

**Objectives:** The aim of this study was to evaluate the psychometric properties of the Pain Catastrophizing Scale (PCS) in patients with carpometacarpal osteoarthritis of the thumb. **Methods:** In this cross-sectional register-based study of 253 patients with carpometacarpal osteoarthritis of the thumb, a two-parameter item response theory analysis was used to evaluate the items’ difficulty and discrimination parameters. **Results:** Of 253 patients, 245 (57%) were women. The mean age was 56.0 (SD 16.5) years. The mean total PCS score was 14.0 (SD 10.5) points. Difficulty estimates were distributed fairly evenly across the item score scale, with a slight shift towards higher scores. Discrimination of both total and subscale scores was perfect, varying from 1.91 to 2.84. **Conclusions:** PCS was able to discriminate well between different levels of catastrophizing. PCS performed slightly more accurately when the catastrophizing level was above average in the studied sample. PCS can be recommended for clinical use when assessing catastrophizing in patients with carpometacarpal osteoarthritis of the thumb.

## 1. Introduction

Carpometacarpal osteoarthritis of the thumb (CMC OA) is a common degenerative condition causing pain and disability [1]. The thumb CMC joint enables the grasp and pinch strength, and because these weaken in CMC OA, the function of the entire hand is impaired [2]. This can lead to a decrease in work and functional capacity, causing psychological strain. In osteoarthritis, the nature and intensity of pain can vary over time, and this unpredictability increases the burden associated with pain [3]. In CMC OA, the treatment outcome and pain perception may be affected by psychological factors such as depression, anxiety, and pain catastrophizing [4,5,6,7]. Pain catastrophizing is defined as a negative attitude toward experienced or anticipated pain. The Pain Catastrophizing Scale (PCS) is a widely used patient-reported outcome measure (PROM) developed by Sullivan et al. in 1995 to assess the level of pain catastrophizing in diverse situations. The PCS contains 13 items divided into three interrelated domains: rumination, magnification, and helplessness [8]. The PCS produces a total score and three subscale scores. In classical test theory, the PCS has been studied in diverse musculoskeletal pain conditions [9,10,11,12,13,14,15]. In a meta-analysis, the PCS has been shown to have good test–retest reliability and good internal consistency for both the total score and the subscales [16]. The reported factor structure of the PCS has varied from one to three factors [8,17,18]. Mixed results have been reported regarding the floor and ceiling effects of the PCS [9,11,12,14,19,20].

Item response theory (IRT) and Rasch analysis are used to assess the effect of the latent factor, here pain catastrophizing, on how the subject responds to a PROM and how well individual items are able to discriminate the level of this latent factor. The psychometric properties of the PCS have previously been evaluated using IRT in chronic pain [21,22], musculoskeletal pain [23,24], low back pain [25,26,27], carpal tunnel syndrome [28], and work-related pain conditions [29]. In these studies, PCS has been found to be suitable for clinical use in the assessment of pain catastrophizing. A shift towards higher scores has been found in low back pain [26] and carpal tunnel syndrome [28]. Differential item functioning (DIF) has been assessed in a few studies [22,27,29]. DIF was not observed in work-related pain conditions [29] or in low back pain [27]. In chronic pain, DIF by sex has been observed for two items [22]. The Rasch analysis has been used in CMC OA in one study that assessed the construct validity of the Measure of Activity Performance of the Hand [30]. To our knowledge, the psychometric properties of the PCS have not been investigated using IRT in CMC OA. Such an assessment is needed before the PCS can be recommended for clinical use in this particular health condition. The aim of this study was to evaluate the psychometric properties of the PCS in CMC OA using IRT.

## 2. Methods

This was a cross-sectional register-based study. The study sample was derived from a register kept by the Wellbeing Services County of Southwest Finland. The ethics of the study have been approved by the research department of Wellbeing Services County of Southwest Finland (2024-11208-TL). According to Finnish law, retrospective register-based research does not require an explicit informed consent from a participant. The data were provided to the research group in an anonymous form without any individually identifiable information. Before each visit to the hand surgery unit of a university hospital, patients received a survey link through which they answered questions regarding demographic information, pain, and functioning. The registry contains data on 14,095 patients. Of these, 350 had a diagnosis of CMC OA (M18.0) as a main diagnosis. Of them, 253 (72%) responded to the PCS questionnaire between 25 April 2019 and 15 May 2024. The methods used for these register data have also been described somewhere else [28].

All patients who responded to the PCS questions during this period were included in the analysis. The registry data did not allow for the possibility of controlling for the characteristics of patients who did not respond to the questionnaire compared to those who did respond. The number of variables available from the registry data was limited, and there was no information to analyze regarding the respondents’ possible comorbidities, for example, possible inflammatory joint disease, pain duration, or previous surgical intervention in the affected limb. Also, bilateral symptoms could not be distinguished from unilateral ones based on the available register data. Radiographic examinations were usually performed before the patient was referred from primary care to a specialist outpatient clinic. Therefore, radiographic findings were not part of the registry in question.

Sex was defined as women vs. men. Dominant hand was defined as right, left, or ambidextrous. Age was defined in full years at the time of response. Body mass index (BMI) was defined as weight divided by height squared (kg/m^2^).

The QuickDASH questionnaire was used to evaluate the limitations in different everyday activities caused by the symptoms in the upper extremity. The QuickDASH contains 11 items assessed on a Likert-like scale from one (no limitation) to five (most severe limitation). The total score was calculated as ([(sum of n responses)/n] − 1) × 25, where “n” is the number of completed responses. The QuickDASH score was considered missing if there was more than one missing item response. The total score of QuickDASH ranges from 0 (no disability) to 100 (most severe disability). QuickDASH was included mainly to describe the sample from a slightly different perspective of functioning.

The PCS assesses the tendency to magnify the treat value of the pain stimulus and to feel helpless because of pain. The scale consists of 13 items graded on a Likert-like scale from zero (not at all) to four (all the time). Additionally, for a total composite score, the PCS produces scores for its three subscales: rumination, magnification, and helplessness. The sub-scores are the sums of responses to items 8, 9, 10, and 11 (rumination); 6, 7, and 13 (magnification); and 1, 2, 3, 4, 5, and 12 (helplessness), respectively. The overall score is the sum of all 13 item scores ranging from 0 to 52 points, with higher scores indicating a greater degree of pain catastrophizing. A total score of >30 points is usually considered a clinically significant level of pain catastrophizing.

### Statistical Analyses

Two-parameter IRT analysis was applied. “Difficulty” describes the perceived severity of catastrophizing needed to obtain a particular score. In other words, the difficulty parameter answers the question “how difficult must a respondent experience pain catastrophizing before he or she ticks a certain answer option?”. Ideally, a respondent will tick the option in the middle of the scale when the level of catastrophizing corresponds to the average level of the studied data. If so, then the difficulty estimates of the IRT analysis carry a minus sign when the perceived severity of catastrophizing is milder than the average and a plus sign when the respondent experiences worse catastrophizing compared to the sample average. In addition, in this ideal situation, the difficulty estimates are distributed evenly across the scale so that each step up or down on the scale represents approximately equal differences in the difficulty of the question. This uniformity is also expected to be seen graphically. The power calculation was not conducted, as it is less critical when the goal is to describe the entire population (here, the entire set of patients is referred to a unit because of CMC OA.

“Discrimination” is the ability of PCS to differentiate people with different levels or measurable entities (here, the level of catastrophizing). Discrimination answers the question: “in which range of catastrophizing severity is the PCS best able to distinguish the level of catastrophizing of respondents?”. Discrimination is the steepness of the regression curve. In plain words, in an ideal situation, the PCS’s discrimination ability should be close to the average level of catastrophizing of the sample. Graphically, we hope to see the steepest part of the curve around the zero value of the *X*-axis (the average level of the sample). If this is the case, then the PCS is able to distinguish respondents whose catastrophizing severity is slightly milder or slightly more severe than the average level, and the test can then be used, for example, as a screening tool. Discrimination of 0.01–0.24 was considered “none” (a totally level regression curve), 0.25–0.64 was considered “low,” 0.65–1.34 was considered “moderate,” 1.35–1.69 was considered “high,” and a discrimination >1.7 was considered “perfect” (a regression curve approaching a vertical line) [31].

Difficulty and discrimination estimates were presented both numerically (accompanied by their 95% CIs) and graphically. The accuracy of the information obtained from the responses to different items was examined through item information functions. Item information function curves were constructed and analyzed graphically. All the analyses were conducted using Stata/IC Statistical Software: Release 18, College Station, (StataCorp LP, College Station, TX, USA).

## 3. Results

The PCS was completed by 253 respondents (Table 1). Of these, 108 (43%) were men and 145 (57%) were women. The mean age was 56.0 (SD 16.5) years. The mean total PCS score was 14.0 (SD 10.5) points. The mean subscale scores were 5.0 (SD 3.8) points for rumination, 3.0 (SD 2.5) points for magnification, and 6.0 (SD 5.0) points for helplessness, respectively. Clinically significant catastrophizing (PCS score > 30 points) was present in 20 (8%) patients.

### 3.1. Difficulty Parameter

**Figure 1:**
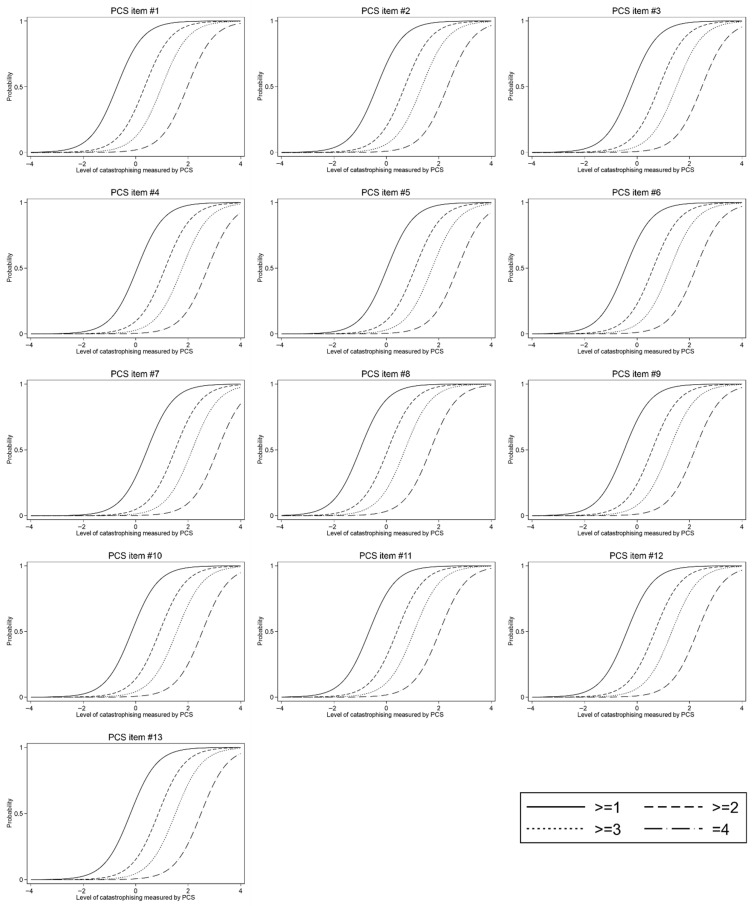
Item response theory analysis—item characteristic curves for all PCS items.

As can be seen in Table 2, for all 13 items, the same phenomenon was observed—estimates with a minus sign appear at the very bottom of the scale, if at all (there was no minus sign at all for items #4 and #5). This suggests that respondents underestimate the severity of their catastrophizing. Respondents ticked the lower options on the scale even when the level of their catastrophizing was higher than the sample mean. However, this tendency was relatively mild for most items. As Figure 1 shows, the distribution of the level of catastrophizing across the scale was not entirely even. For all items, it was seen that the distance between the values “2” and “3” in the middle of the scale was slightly narrower (smaller) compared to the other answer options.

### 3.2. Discrimination Parameter

**Figure 2:**
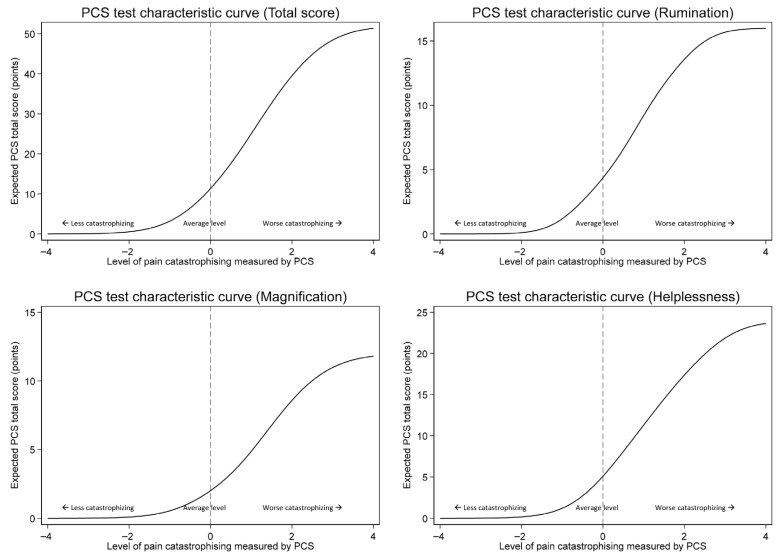
Item response theory analysis—test characteristic curves for PCS total score and subscales.

The discrimination of both the PCS total and subscale scores was perfect (Table 3). The discrimination of the PCS total score was 1.99 (95% CI 1.76–2.21). The discrimination of the subscales was 2.84 (95% CI 2.41–3.27) for rumination, 1.91 (95% CI 1.55–2.28) for magnification, and 2.11 (95% CI 1.82–2.39) for helplessness. The test characteristic curves for the PCS total score and subscale scores were steepest at slightly higher than average levels of pain catastrophizing (Figure 2).

### 3.3. Item Information Function

**Figure 3:**
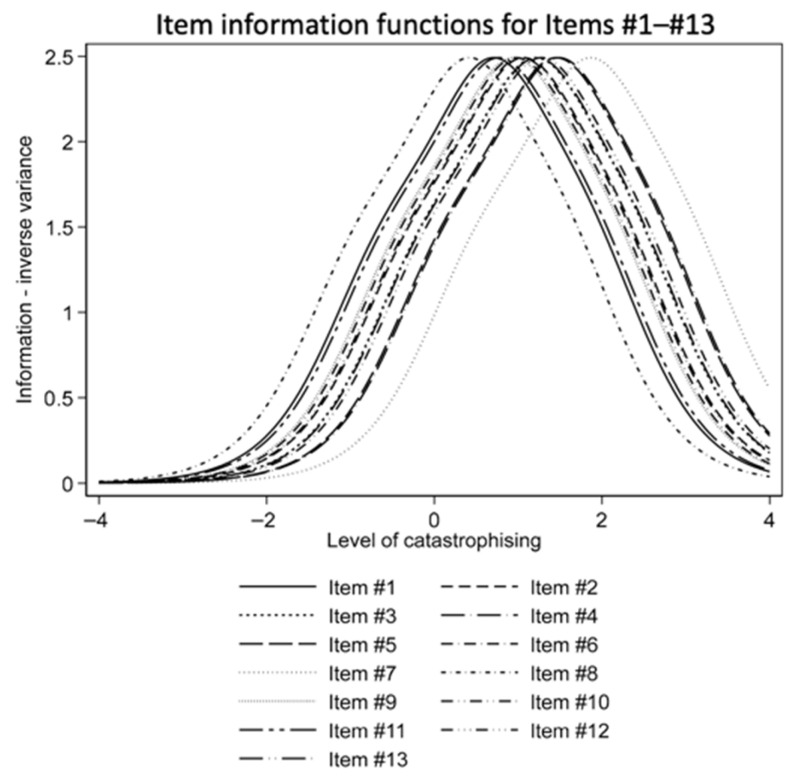
Item information function for all PCS items.

As can be seen in Figure 3, the item information function curves were almost identical for all items. All curves were shifted to the right from the zero point on the *X*-axis (the mean level of catastrophizing severity for this sample). This means that the information obtained from the item responses was more reliable (less variable) when the level of catastrophizing was higher compared to the sample mean.

## 4. Discussion

It has been previously reported that psychological factors, such as pain catastrophizing, can affect treatment outcomes and pain experience in CMC OA [4,5,6,7]. In this study, a two-parameter IRT analysis was used to evaluate the properties of the PCS amongst 253 patients with CMC OA. Overall, the PCS seemed to work quite well in the studied sample. However, some deviations in its operation were observed. The ability to obtain reliable information from the answers to the PCS questions was found to be slightly above the average level of catastrophizing. The information obtained from the item responses was more reliable (less variable) when the level of catastrophizing was higher compared to the sample mean. A shift in the discrimination parameter to the right (high difficulty) means that the answer to the item is relatively difficult. This means that people with a high level of latent trait (here, catastrophizing) in particular have a high probability of answering correctly. The clinician can expect to gain a somewhat clearer understanding of the patient’s level of catastrophizing when the patient experiences more severe than average catastrophizing. For an individual patient, these deviations were quite mild, and their impact on the clinical use of the PCS is probably minor. And they may not usually be taken into account in routine use. Examination of the item difficulty parameter showed a slight underestimation by the respondents of their level of catastrophizing. In addition, the distribution of the response options on the item scale according to difficulty was slightly uneven. This unevenness, which is shown in Figure 2, may require modification of the PCS scoring system, but only if the need is to obtain a particularly accurate picture of the level of catastrophizing, for example, in the context of scientific research. Changing the established scoring formula always leads to an increase in the threshold for using the test and difficulties in terms of the comparability of results. This unevenness was so mild that there is no reason to adapt the commonly accepted scoring system to it in routine clinical use. The discrimination estimates suggested that the PCS works best when the level of catastrophizing is slightly above the average level. In other words, the PCS is particularly accurate in distinguishing respondents who have clearly more severe catastrophizing from those who have slightly more severe catastrophizing relative to the population average. In the range of milder levels of catastrophizing, the measure is less accurate.

The properties of the PCS have not been previously assessed amongst patients with CMC OA. Thus, the present results cannot be directly compared to previous knowledge. On the other hand, the properties of the PCS have been previously investigated using IRT analysis in several other, but different, chronic pain conditions [22,23,24,25,26,27,28,29]. The present results were in line with the reports on the properties of PCS in other disorders than CMC OA. A shift towards higher scores has also been observed before amongst patients with low back pain [26] and in carpal tunnel syndrome [28].

The generalizability of the results of this study could be weakened by several factors. The data have been collected in a single university hand surgery unit. The symptoms and level of catastrophizing of patients with CMC OA referred to a highly specialized unit are likely to be more severe than in primary care. This was a register-based study without access to missing data. Therefore, as is common in register-based research, the effect of unmeasured confounding factors, such as medication and comorbidities, might have affected the results. It is possible that unknown comorbidities, previous surgeries in the limb in question, or unilateral or bilateral symptomatology may have influenced the results. However, this is unlikely, as the focus of the study was the psychometric properties of the questionnaire and not the level of catastrophizing in the sample studied. Also, the inability to assess missing data could have been a source of systematic bias. And this risk must be taken into account when drawing conclusions from these results. Furthermore, unlike classical test theory (where the difficulty of a question depends on the individual characteristics of the respondent), one of the strengths of IRT is its invariance. The parameters of a question can, therefore, be estimated from any point on the response curve of a question. This allows for the comparability of responses, even if the respondents belong to different groups or have answered different sets of questions. In the case of IRT, the characteristics of the test are not “trapped” in a specific sample group or set of questions. In other words, even if the level of catastrophizing was affected by unknown covariates, this difference in level does not affect the results of the IRT analysis. Of course, the group of respondents should have some clear categorizing common characteristic—in this case, the patients have had CMC OA—and this was the main reason for answering the questionnaire. The invariance of IRT corresponds in principle to the invariance of the Rasch analysis. The sample was predominated by woman, which was expected since CMC OA is more common in women [1]. In one study amongst patients with chronic pain, DIF by sex was found for two items [22]. However, no DIF has been observed in work-related pain conditions [29] or in people with chronic low back pain [27]. The sample included patients of a relatively limited age group of <60 years, and the severity of disability (measured by using QuickDASH) was relatively mild. The prevalence of CMC OA increases with age [1]. It is possible that the results could differ if the PCS was applied to an older age group, where OA and other diseases causing disability are more common. The limitations in upper-limb functioning may be more likely to increase catastrophizing if the lifestyle is already sedentary due to older age and retirement. On the other hand, in a working-age population, the possible decrease in ability to work caused by CMC OA might increase financial problems and, in this way, worsen catastrophizing.

Further research on the psychometric properties of the PCS may be useful. Since DIF has only been mentioned in a few studies of the PCS, further investigation could provide more information on whether different factors, such as sex or age, affect pain catastrophizing in patients with CMC OA. DIF analysis is such a broad topic (if we take into account several categorizing factors) that it deserves its own separate study. The results of this study should be replicated in different settings as well.

## 5. Conclusions

The PCS was able to discriminate well between different levels of catastrophizing. PCS performed slightly more accurately when the catastrophizing level was above average in the studied sample. PCS can be recommended for clinical use when assessing catastrophizing in patients with carpometacarpal osteoarthritis of the thumb.

## Figures and Tables

**Table 1:** Descriptive characteristics of sample—*n* (%) or mean (standard deviation).

Characteristic	Summary
n	253
Sex	
Men	108 (43%)
Women	145 (57%)
Dominant hand	
Right	116 (94%)
Left	6 (5%)
Ambidextrous	2 (2%)
Age, years	56 (16.5)
Body mass index, kg/m^2^	28 (5.6)
QuickDASH, points	38 (22.6)
Presence of clinically significant catastrophising ^a^	
No	233 (92%)
Yes	20 (8%)
PCS	
Total score	14 (10.5)
Rumination	5 (3.8)
Magnification	3 (2.5)
Helplessness	6 (5.0)

^a^ PCS score ≤30 vs. >30 points.

**Table 2:** Item response theory analysis—difficulty parameter of PCS items.

PCS Item	Coefficient	95% CI			PCS Item	Coefficient	95% CI	
Item #1 I worry all the whether the pain will end					Item #8 I anxiously want the pain to go away			
1 vs. 0	−0.72	−0.90	−0.54		1 vs. 0	−1.01	−1.20	−0.82
2 vs. 1	0.34	0.18	0.51		2 vs. 1	0.05	−0.11	0.22
3 vs. 2	0.99	0.80	1.18		3 vs. 2	0.70	0.52	0.88
4 vs. 3	1.97	1.69	2.25		4 vs. 3	1.68	1.42	1.94
Item #2 I feel I can’t go on					Item #9 I can’t seem to keep it out of my mind			
1 vs. 0	−0.36	−0.53	−0.20		1 vs. 0	−0.49	−0.66	−0.32
2 vs. 1	0.70	0.52	0.88		2 vs. 1	0.57	0.40	0.75
3 vs. 2	1.35	1.14	1.56		3 vs. 2	1.23	1.02	1.43
4 vs. 3	2.33	2.02	2.63		4 vs. 3	2.20	1.91	2.50
Item #3 It’s terrible and I think it’s never going to get any better					Item #10 I keep thinking about how much it hurts			
1 vs. 0	−0.22	−0.38	−0.05		1 vs. 0	−0.15	−0.32	0.01
2 vs. 1	0.84	0.66	1.03		2 vs. 1	0.91	0.72	1.10
3 vs. 2	1.49	1.27	1.72		3 vs. 2	1.56	1.33	1.79
4 vs. 3	2.47	2.15	2.79		4 vs. 3	2.54	2.22	2.86
Item #4 It’s awful and I feel it over whelms me					Item #11 I keep thinking about how badly I want the pain to stop			
1 vs. 0	0.06	−0.11	0.22		1 vs. 0	−0.66	−0.84	−0.49
2 vs. 1	1.12	0.92	1.32		2 vs. 1	0.40	0.24	0.57
3 vs. 2	1.77	1.52	2.01		3 vs. 2	1.05	0.86	1.25
4 vs. 3	2.75	2.40	3.09		4 vs. 3	2.03	1.75	2.31
Item #5 I feel I can’t stand it anymore					Item #12 There’s nothing I can do to reduce the intensity of the pain			
1 vs. 0	0.03	−0.14	0.19		1 vs. 0	−0.39	−0.56	−0.22
2 vs. 1	1.09	0.89	1.29		2 vs. 1	0.67	0.50	0.85
3 vs. 2	1.74	1.50	1.98		3 vs. 2	1.33	1.11	1.54
4 vs. 3	2.72	2.38	3.05		4 vs. 3	2.30	2.00	2.61
Item #6 I become afraid that the pain will get worse					Item #13 I wonder whether something serious might happen			
1 vs. 0	−0.45	−0.62	−0.28		1 vs. 0	−0.20	−0.37	−0.04
2 vs. 1	0.61	0.44	0.79		2 vs. 1	0.86	0.67	1.04
3 vs. 2	1.26	1.06	1.47		3 vs. 2	1.51	1.29	1.74
4 vs. 3	2.24	1.94	2.54		4 vs. 3	2.49	2.17	2.81
Item #7 I keep thinking of other painful events								
1 vs. 0	0.44	0.26	0.61					
2 vs. 1	1.50	1.27	1.73					
3 vs. 2	2.15	1.87	2.43					
4 vs. 3	3.13	2.75	3.50					

**Table 3:** Item response theory analysis—discrimination parameter of PCS and its subscales.

Discrim	Coefficient	95% CI	
Rumination	2.84	2.41	3.27
Magnification	1.91	1.55	2.28
Helplessness	2.11	1.82	2.39
Total score	1.99	1.76	2.21

## Data Availability

Data are available from the corresponding author on a reasonable request.

## References

[1] Haara M.M., Heliövaara M., Kröger H., Arokoski J.P., Manninen P., Kärkkäinen A., Knekt P., Impivaara O., Aromaa A. (2004). Osteoarthritis in the carpometacarpal joint of the thumb. Prevalence and associations with disability and mortality. J. Bone Jt. Surg. Am..

[2] Ladd A.L., Weiss A.P., Crisco J.J., Hagert E., Wolf J.M., Glickel S.Z., Yao J. (2013). The thumb carpometacarpal joint: Anatomy, hormones, and biomechanics. Instr. Course Lect..

[3] Hawker G.A., Stewart L., French M.R., Cibere J., Jordan J.M., March L., Suarez-Almazor M., Gooberman-Hill R. (2008). Understanding the pain experience in hip and knee osteoarthritis—An OARSI/OMERACT initiative. Osteoarthr. Cartil..

[4] Hoogendam L., van der Oest M.J.W., Tsehaie J., Wouters R.M., Vermeulen G.M., Slijper H.P., Selles R.W., Porsius J.T. (2021). Psychological factors are more strongly associated with pain than radiographic severity in non-invasively treated first carpometacarpal osteoarthritis. Disabil. Rehabil..

[5] Hoogendam L., van der Oest M.J.W., Wouters R.M., Andrinopoulou E.R., Vermeulen G.M., Slijper H.P., Porsius J.T., Selles R.W. (2021). Patients with Higher Treatment Outcome Expectations Are More Satisfied with the Results of Nonoperative Treatment for Thumb Base Osteoarthritis: A Cohort Study. Arch. Phys. Med. Rehabil..

[6] Mulrooney E., Neogi T., Dagfinrud H., Hammer H.B., Pettersen P.S., Gaarden T.L., Engedal K., Kvien T.K., Magnusson K., Haugen I.K. (2022). The associations of psychological symptoms and cognitive patterns with pain and pain sensitization in people with hand osteoarthritis. Osteoarthr. Cartil. Open.

[7] Wouters R.M., Vranceanu A.M., Slijper H.P., Vermeulen G.M., van der Oest M.J.W., Selles R.W., Porsius J.T. (2019). Patients with Thumb-base Osteoarthritis Scheduled for Surgery Have More Symptoms, Worse Psychological Profile, and Higher Expectations Than Nonsurgical Counterparts: A Large Cohort Analysis. Clin. Orthop. Relat. Res..

[8] Sullivan M.J.L., Bishop S.R., Pivik J. (1995). The pain catastrophizing scale: Development and validation. Psychol. Assess..

[9] Mikkonen J., Leinonen V., Lähdeoja T., Holopainen R., Ekström K., Koho P., Airaksinen O., Luciano J.V., Navarrete J., Neblett R. (2024). Dimensionality, reliability, and validity of the Finnish version of the pain catastrophizing scale in chronic low back pain. Scand. J. Pain.

[10] Van Damme S., Crombez G., Bijttebier P., Goubert L., Van Houdenhove B. (2002). A confirmatory factor analysis of the Pain Catastrophizing Scale: Invariant factor structure across clinical and non-clinical populations. Pain.

[11] İlçin N., Gürpınar B., Bayraktar D., Savcı S., Çetin P., Sarı İ., Akkoç N. (2016). Cross-cultural adaptation and validation of the Turkish version of the pain catastrophizing scale among patients with ankylosing spondylitis. J. Phys. Ther. Sci..

[12] Ong W.J., Kwan Y.H., Lim Z.Y., Thumboo J., Yeo S.J., Yeo W., Wong S.B., Leung Y.Y. (2021). Measurement properties of Pain Catastrophizing Scale in patients with knee osteoarthritis. Clin. Rheumatol..

[13] Chibnall J.T., Tait R.C. (2005). Confirmatory factor analysis of the Pain Catastrophizing Scale in African American and Caucasian Workers’ Compensation claimants with low back injuries. Pain.

[14] Fernandes L., Storheim K., Lochting I., Grotle M. (2012). Cross-cultural adaptation and validation of the Norwegian pain catastrophizing scale in patients with low back pain. BMC Musculoskelet. Disord..

[15] Kemani M.K., Grimby-Ekman A., Lundgren J., Sullivan M., Lundberg M. (2019). Factor structure and internal consistency of a Swedish version of the Pain Catastrophizing Scale. Acta Anaesthesiol. Scand..

[16] Wheeler C.H.B., Williams A.C.C., Morley S.J. (2019). Meta-analysis of the psychometric properties of the Pain Catastrophizing Scale and associations with participant characteristics. Pain.

[17] Osman A., Barrios F.X., Kopper B.A., Hauptmann W., Jones J., O’Neill E. (1997). Factor structure, reliability, and validity of the Pain Catastrophizing Scale. J. Behav. Med..

[18] Bascour-Sandoval C., Albayay J., Martínez-Molina A., Opazo-Sepúlveda A., Lacoste-Abarzúa C., Bielefeldt-Astudillo D., Gajardo-Burgos R., Galvéz-García G. (2022). Psychometric Properties of the PCS and the PCS-4 in Individuals with Musculoskeletal Pain. Psicothema.

[19] Kibet J.J., Phillips J.S., Latrous M.C., Khalil H., Morris L.D. (2024). Translation, cultural adaptation and validation of the Swahili Pain Catastrophizing Scale among refugees who survived torture and/or war trauma in Kenya: An observational study. Health Sci. Rep..

[20] Ceniza-Bordallo G., Gómez Fraile A., Martín-Casas P., López-de-Uralde-Villanueva I. (2023). Validation of the Spanish version of the Pain Catastrophizing Scale for Children (PCS-C). An. Pediatría (Engl. Ed.).

[21] Amtmann D., Jensen M., Turk D., Lavallee D., Liljenquist K., Bamer A., Salem R. (2019). PCORI Final Research Reports. Developing Measures of Pain Appraisal and Pain-Related Self-Efficacy for People Living with Chronic Pain.

[22] Walton D.M., Mehta S., Seo W., MacDermid J.C. (2020). Creation and validation of the 4-item BriefPCS-chronic through methodological triangulation. Health Qual. Life Outcomes.

[23] Nishigami T., Mibu A., Tanaka K., Yamashita Y., Watanabe A., Tanabe A. (2017). Psychometric properties of the Japanese version of short forms of the Pain Catastrophizing Scale in participants with musculoskeletal pain: A cross-sectional study. J. Orthop. Sci..

[24] Le Carré J., Luthi F., Burrus C., Konzelmann M., Vuistiner P., Léger B., Benaïm C. (2023). Development and Validation of Short Forms of the Pain Catastrophizing Scale (F-PCS-5) and Tampa Scale for Kinesiophobia (F-TSK-6) in Musculoskeletal Chronic Pain Patients. J. Pain Res..

[25] Lopes R.A., Dias R.C., Queiroz B.Z., Rosa N.M., Pereira Lde S., Dias J.M., Magalhães Lde C. (2015). Psychometric properties of the Brazilian version of the Pain Catastrophizing Scale for acute low back pain. Arq. Neuro-Psiquiatr..

[26] Franchignoni F., Giordano A., Ferriero G., Monticone M. (2022). Measurement precision of the Pain Catastrophizing Scale and its short forms in chronic low back pain. Sci. Rep..

[27] Meroni R., Piscitelli D., Bonetti F., Zambaldi M., Cerri C.G., Guccione A.A., Pillastrini P. (2015). Rasch Analysis of the Italian version of Pain Catastrophizing Scale (PCS-I). J. Back. Musculoskelet. Rehabil..

[28] Saltychev M., Miikkulainen A., Taskinen H.S. (2025). Properties of pain catastrophizing scale amongst patients with carpal tunnel syndrome—Item response theory analysis. Scand. J. Pain.

[29] Walton D.M., Wideman T.H., Sullivan M.J. (2013). A Rasch analysis of the pain catastrophizing scale supports its use as an interval-level measure. Clin. J. Pain.

[30] Tveter A.T., Østerås N., Nossum R., Eide R.E., Klokkeide Å., Matre K.H., Olsen M., Andreassen Ø., Hammond A., Kjeken I. (2020). The MAP-Hand: Psychometric properties and differences in activity performance between patients with carpometacarpal osteoarthritis and rheumatoid arthritis. J. Rehabil. Med..

[31] Baker F.B. (2001). The Basics of Item Response Theory.

